# Expression of the ZIP/*SLC39A* transporters in β-cells: a systematic review and integration of multiple datasets

**DOI:** 10.1186/s12864-017-4119-2

**Published:** 2017-09-11

**Authors:** Rebecca Lawson, Wolfgang Maret, Christer Hogstrand

**Affiliations:** King’s College London, Faculty of Life Sciences and Medicine, Diabetes and Nutritional Sciences, Metal Metabolism Group, 150 Stamford St, London, SE1 9NH UK

**Keywords:** Type 2 diabetes, Zinc, ZIP, *SLC39A*, Systematic review, Expression data, Microarray, RNA-seq

## Abstract

**Background:**

Pancreatic β-cells require a constant supply of zinc to maintain normal insulin secretory function. Following co-exocytosis with insulin, zinc is replenished via the Zrt- and Irt-like (ZIP; *SLC39A*) family of transporters. However the ZIP paralogues of particular importance for zinc uptake, and associations with β-cell function and Type 2 Diabetes remain largely unexplored. We retrieved and statistically analysed publically available microarray and RNA-seq datasets to perform a systematic review on the expression of β-cell *SLC39A* paralogues. We complemented results with experimental data on expression profiling of human islets and mouse β-cell derived MIN6 cells, and compared transcriptomic and proteomic sequence conservation between human, mouse and rat.

**Results:**

The 14 ZIP paralogues have 73–98% amino sequence conservation between human and rodents. We identified 18 datasets for β-cell *SLC39A* analysis, which compared relative expression to non-β-cells, and expression in response to PDX-1 activity, cytokines, glucose and type 2 diabetic status. Published expression data demonstrate enrichment of transcripts for ZIP7 and ZIP9 transporters within rodent β-cells and of ZIP6, ZIP7 and ZIP14 within human β-cells, with ZIP1 most differentially expressed in response to cytokines and PDX-1 within rodent, and ZIP6 in response to diabetic status in human and glucose in rat. Our qPCR expression profiling data indicate that *SLC39A6, −9, −13, and − 14* are the highest expressed paralogues in human β-cells and *Slc39a6* and *−7* in MIN6 cells.

**Conclusions:**

Our systematic review, expression profiling and sequence alignment reveal similarities and potentially important differences in ZIP complements between human and rodent β-cells. We identify ZIP6, ZIP7, ZIP9, ZIP13 and ZIP14 in human and rodent and ZIP1 in rodent as potentially biologically important for β-cell zinc trafficking. We propose ZIP6 and ZIP7 are key functional orthologues in human and rodent β-cells and highlight these zinc importers as important targets for exploring associations between zinc status and normal physiology of β-cells and their decline in Type 2 Diabetes.

**Electronic supplementary material:**

The online version of this article (10.1186/s12864-017-4119-2) contains supplementary material, which is available to authorized users.

## Background

Pancreatic β-cells require a constant supply of zinc for normal function in maintaining glycaemic control [1, 2]. Zinc acts at multiple stages within the insulin secretory pathway [3, 4]. Zinc ions (Zn^2+^) are loaded into insulin granules via the predominantly β-cell specific zinc transporter 8 (ZnT8) [5], where two ions co-crystallise with insulin hexamers [6], important for proper insulin processing, protection of insulin from proteolytic degradation [7] and for maintaining granule osmotic stability [8]. Zinc is subsequently co-released with mature insulin upon exocytosis where it is proposed to fulfil additional roles in glycaemic control [9–11].

Significant amounts of Zn^2+^ are lost from β-cells during insulin secretion and coordinated replenishment is required. The Zrt- and Irt-like (ZIP; *SLC39A*) family of zinc importer proteins, of which 14 paralogues are present within both humans and rodents [12, 13], tightly control cellular Zn^2+^ influx into the cytosol and are thought responsible for restoring β-cell zinc content [14]. ZIP paralogues exhibit differing Zn^2+^ affinities (K_0.5_) and transporting efficiencies, and show cell- and condition-dependent expression [12, 15], thus it is expected that the β-cell ZIP profile closely reflects the unique cellular demand for Zn^2+^ and ability to adapt to stresses such as hyperglycaemia and inflammatory cytokines. Since both hyperzincemic and hypozincemic Zn^2+^statuses are observed in diabetic patients [16–18] and animal models of diabetes [19, 20], one can hypothesize that altered ZIP expression profiles are associated with disease state. However exploration of the β-cell *SLC39A* transcriptome, and therefore the liable transporters, has been limited to a few studies [4, 14, 21–23], where an importance of ZIP4 [23], ZIP6 [21, 22], ZIP7 [14, 21, 22], ZIP8 [22], and ZIP14 [14, 24] has been suggested.

Type 2 Diabetes is rapidly evolving into a major public health crisis. The disease pathogenesis generally results from an increasingly inadequate insulin response due to enhanced insulin resistance and a compensatory demand on insulin production that eventually leads to β-cell failure. Multiple studies have associated diabetes with hypozincemia, likely caused by hyperzincuria, and a negative correlation between the glycated haemoglobin percentage and plasma zinc [16–18]. Accordingly, there is a positive effect of adequate plasma zinc levels on glycemic control [18], suggesting a compromised zinc status in diabetes [25].

Since zinc plays an integral role within β-cells, understanding its regulation may prove central for targeting loss of secretory function during Type 2 Diabetes. Much of our understanding of β-cell physiology has derived from studies on rodents due to very limited accessibility of human islets [26]. However, differences in physiology between humans and rodents remain often unacknowledged when interpreting rodent studies. We hypothesised that the ZIP transporters most important to β-cells should be robustly expressed and show enrichment relative to other cell types [27], with changes in expression influenced by cellular stresses associated with compromised insulin secretion. We thereby aimed to identify and evaluate the complement of ZIP transporters most important within human and rodent (mouse and rat) β-cells for regulating zinc influx and accumulation.

Here we show through systematic review of microarray and RNA-seq studies [28, 29] that transcripts for multiple ZIP paralogues are enriched in β-cells and/or show transcriptional regulation in response to cytokines, hyperglycaemia, Type 2 Diabetes status, and pancreatic and duodenal homeobox 1 (PDX-1) activity, the major transcription factor for β-cells. We used quantitative PCR (qPCR) to verify the relative expression of these paralogues within human islets and/or murine MIN6 β-cells. Furthermore, we computationally aligned human, mouse and rat SLC39A mRNA and protein sequences to demonstrate high cross-species conservation of the paralogues identified as key for β-cell zinc homeostasis within our systematic review. We highlight ZIP6, ZIP7, ZIP9, ZIP13 and ZIP14 in human and rodent, and ZIP1 in rodent as biologically important candidates for mediating β-cell Zn^2+^ influx and zinc-signalling processes, such as cell proliferation. In addition to normal physiology, we suggest ZIP6, ZIP7 and ZIP14 downregulation is associated with diabetic status; however the relationship to zinc content in the β-cells/pancreas remains unknown. Critically, our review highlights potentially important differences between human islets and rodent cells in their complements of zinc importers, again demonstrating the limitations of rodent models for human diabetes.

## Methods

### Systematic review

#### Identification of eligible expression datasets

**Fig. 1:**
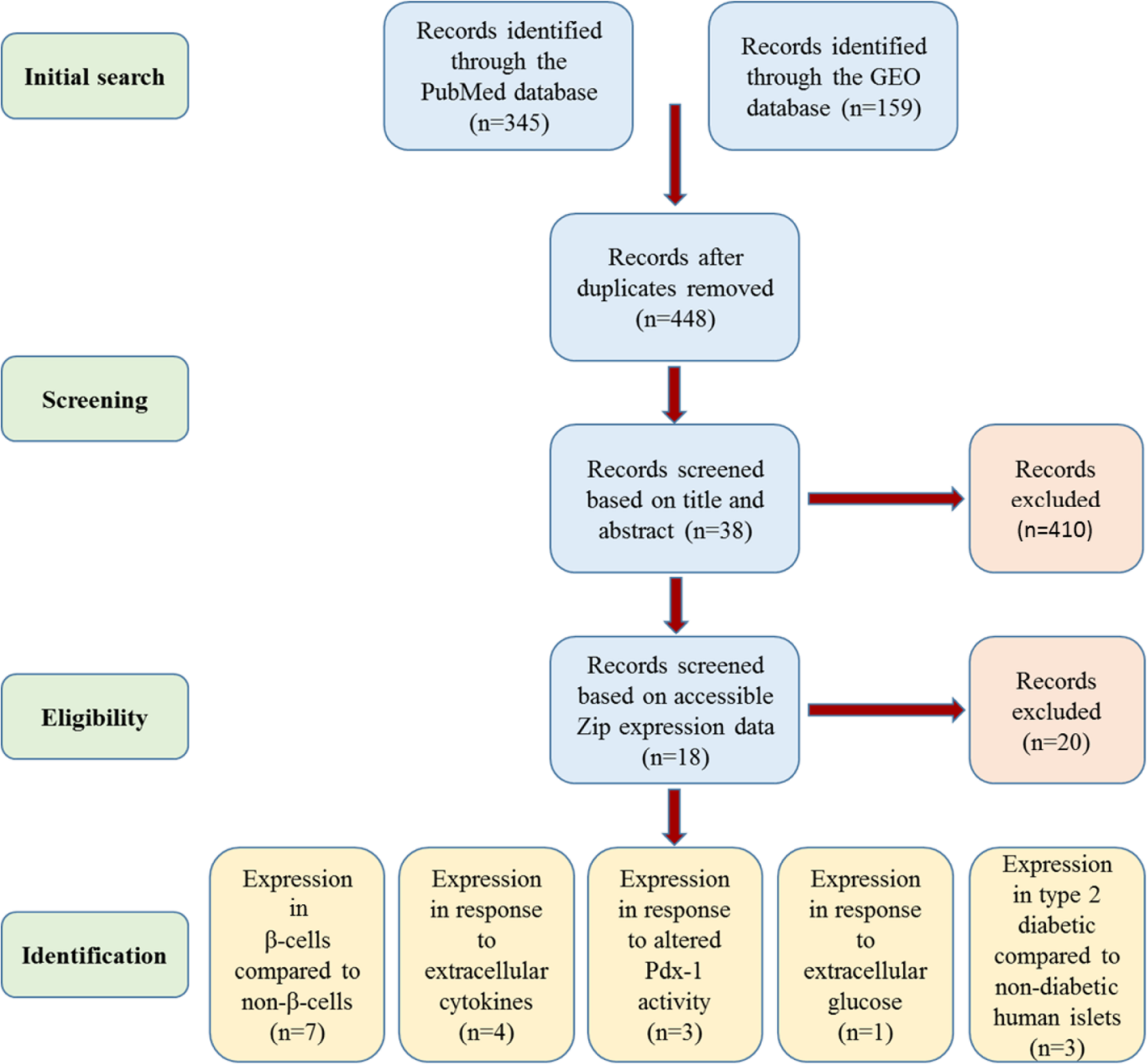
The pipeline followed for identification of suitable microarray and RNA-seq datasets

This systematic review was conducted in accordance with the guidelines provided in the PRISMA statement. Microarray and RNA-seq expression profiling studies were identified through searching the NCBI PubMed database and the Gene Expression Omnibus (GEO) database [30] to April 2016, using combinations of the following key terms: “β-cell, islet” and “diabetes, gene expression, microarray, RNA-seq”, and compiled studies screened for duplicates. Eligibility was independently assessed through first screening by title and abstract, and then by the full text, based on the following inclusion criteria: original research article published in English, RNA-seq or microarray platform, expression profiling of mature β-cells, islets and/or β-cell line, and human or rodent genome. The eligibility was finally confirmed through verifying the presence of accessible expression data for ZIP transporter transcripts (*SLC39A/Slc39a*). Included datasets explored: (a) expression within β-cells compared to non-β-cells, (b) expression in response to extracellular cytokines, (c) expression in response to PDX-1 activity, (d) expression in response to extracellular glucose, and (e) expression within human diabetic islets. From each identified dataset, the accession number (if appropriate), platform, species, sample types and sizes, and gene expression data were extracted. This pipeline is depicted in Fig. 1.

#### Data pre-processing

The heterogeneity of different platforms, gene nomenclature and control samples can cause difficulties when comparing datasets from different sources. Normalisation is therefore critical to reduce the chance of skewing the results and enhances credibility of individual expression changes. To minimise inconsistency, a standardised normalisation method was performed within datasets [31] using Qlucore Omics Explorer (version 3.2; Qlucore AB, Lund, Sweden). Raw data was log (base = 2; log_2_) transformed and normalised through applying a standard score (Z-score) transformation, which calculates normalised expression intensities (y_i) of each probe as follows:

$$ {\mathrm{y}}_{\mathrm{i}}=\frac{{\mathrm{x}}_{\mathrm{i}}-\mathrm{m}}{\updelta} $$

Where x_i represents raw intensity values (x_i, *i* = 0…N-1) for each gene, m represents average gene intensity for the experiment, and δ represents the standard deviation of all measured intensities.

#### Statistical analysis

Statistical analysis was undertaken using the Qlucore Omics Explorer (version 3.2; Qlucore AB, Lund, Sweden) bioinformatics software. Fold differences (FD) in expression between relevant conditions were derived, and significance calculated on the global transcriptomic data set through unpaired t-tests, adjusted using the Benjamini-Hochberg False Discovery Rate (FDR) procedure [32]. Genes were considered differentially expressed in comparisons at an FDR of 15%. Arbitrary FD cut offs of ≥1.5-fold on significantly regulated genes were chosen to indicate biologically relevant differential expression. Full results from analysis are provided in Additional file 1.

#### Datasets with inaccessible raw data

Where the full raw datasets were not available for download, normalised data were extracted from supplementary data tables and log_2_ transformed FD (Log_2_FD) values and significance extracted/calculated using Excel as appropriate. Data analysed in this way are annotated.

### Experimental analysis

#### Human islet cDNA

Human islet cDNA originating from healthy cadaver donors was obtained via the Human Islet Isolation Unit at King’s College Hospital.

#### Cell line and RNA extraction

The adherent insulinoma β-cell line MIN6 (*Mus musculus*) was maintained within Dulbecco’s Modified Eagle’s Medium supplemented with 15% fetal bovine serum, 4 mM L-glutamine, 50 μM β*-*mercaptoethanol, 100 μg/ml streptomycin and 100 units/ml penicillin (both Sigma-Aldrich), at 37 °C in a humidified atmosphere of 95% air and 5% CO_2_. Total RNA was extracted using TRIzol Reagent (ThermoFisher), reverse transcribed to cDNA using the high capacity RNA-to-cDNA kit (ThermoFisher), and diluted ≥1:10 prior to experimentation.

#### Quantitative PCR

Quantitative PCR (qPCR) assays were designed using the online Universal Probe Library (UPL) assay design tool (Roche). Assay designs are provided within Additional files 2 and 3: Tables S1 and S2. Primer Blast [33] was used to predict the binding of our primers to mouse and human RNA. The mouse primers bind all respective ZIP transporter isoforms. The human primers bind all isoforms for ZIP2, ZIP4, ZIP5, ZIP6, ZIP8, ZIP10, ZIP11, ZIP12 and ZIP13. Primers for ZIP1, ZIP3, ZIP7, ZIP9 and ZIP14 covered either all the isoforms expressed within human islets, or those predominantly expressed (Additional files 2 and 3: Tables S1 and S2, Additional file 4). PCR plates were loaded using the Biomek FX liquid handling robot (Beckman Coulter) and reactions [20-40 μg cDNA, 0.1 μM UPL probe, 0.2 μM forward primer, 0.2 μM reverse primer and 1X TaqMan Fast Advanced Mastermix (Applied Biosystems)] amplified using the Prism7900HT sequence detection system, Applied Biosystems, and analysed with SDS (sequence detection systems) 2.4 software. All gene expression values were normalised to the housekeeping gene ubiquitin C (*UBC*), and relative expression calculated using the ΔΔCT method. The efficiencies for primers targeting genes with appreciable expression (mouse: ZIP1, ZIP2, ZIP3, ZIP4, ZIP6, ZIP7, ZIP8, ZIP9, ZIP10, ZIP11, ZIP13, ZIP14, UBC, GADPH; human: ZIP1, ZIP3, ZIP4, ZIP5, ZIP6, ZIP7, ZIP8, ZIP9, ZIP10, ZIP13, ZIP14, UBC, GADPH) ranged from 88 to 111% for mouse and 75–106% for human. Data show an average of two biological repeats for human islets and three biological repeats for MIN6 cells.

#### Exploration of zinc transporter heterogeneity

Human and mouse ZIP orthologues were aligned to assess transcriptomic and proteomic similarities using MUSCLE (3.8) [34, 35] and percent similarity values recorded.

## Results

### Overview of included datasets

A systematic review allows integrated analysis of multiple high throughput gene expression datasets. Following the pre-defined criteria, 18 appropriate β-cell/islet expression profiling studies were identified. These studies are summarised in Table 1. Seven studies compared expression within β-cells to non-β-cells, four investigated expression in response to extracellular cytokines, three studied expression in response to PDX-1 activity, one explored expression in response to extracellular glucose, and three measured expression within human diabetic islets.

**Table 1:** Overview of the datasets identified for analysis

Dataset ID	Platform	Species	Sample	Number of samples	Reference
E-MTAB-463 and E-MTAB-465	Agilent-014850 Whole Human Genome Microarray 4x44K G4112F	*Homo sapiens*	β-cells and α-cells	β-cells: 4 α-cells: 4	Dorrell et al., 2014
–	RNA-seq	*Homo sapiens*	Sorted β-cells, α-cells and exocrine cells (duct and acinar)	β-cells: 3 α-cells: 3 Exocrine cells: 2	Bramswig et al., 2013
EGAS00001000442	RNA-seq	*Homo sapiens*	Sorted β-cells, α-cells and non-β-cells	7 DNA libraries, pooled	Nica et al., 2013
GSE30803	[HG-U133A] Affymetrix Human Genome U133A Array	*Homo sapiens*	Islets (and 16 other primary cell types)	Islets: 3	Martens et al., 2011
–	RNA-seq	*Mus musculus*	β-cells and islets	Unknown. Data for 5 additional cell types downloaded from NCBI	Ku et al., 2012
GSE13381	[Rat230_2] Affymetrix Rat Genome 230 2.0 Array	*Rattus norvegicus*	β-cells and α-cells	β-cells: 2 Non-β-cells: 2	Kutlu et al., 2009
GSE10785	Rosetta/Merck Mouse 44 k 1.0 microarray	*Mus musculus*	Islets (and 5 other primary cell types)	Islets: 40	Keller et al., 2008
GSE35296	RNA-seq	*Homo sapiens*	Islets	Control: 5 IL-1β and IFN-γ: 5	Eizirik et al., 2012
–	[HG-U133A] Affymetrix Human Genome U133A Array	*Homo sapiens*	Islets	Control: 3 IL-1β and IFN-γ: 3	Ylipaasto et al., 2005
–	[Rat230_2] Affymetrix Rat Genome 230 2.0 Array	*Rattus norvegicus*	FACS purified β-cells	For control and cytokine stimulation at each time point: 3	Ortis et al., 2010
–	[Rat230_2] Affymetrix Rat Genome 230 2.0 Array	*Rattus norvegicus*	INS-1E rat insulinoma cell line	For control and cytokine stimulation at each time point: 3	Moore et al., 2011
GSE40642	[Rat230_2] Affymetrix Rat Genome 230 2.0 Array	*Rattus norvegicus*	INS-1ab rat insulinoma cell line	Control: 8 IL-1β: 20 PDX-1 overexpression: 8 PDX-1 overexpression and IL-1β: 20	Hansen et al., 2012
GSE49786	[Rat230_2] Affymetrix Rat Genome 230 2.0 Array	*Rattus norvegicus*	Islets	Untreated: 5 Pdx-1 overexpression: 5	Hayes et al., 2013
E-MTAB-127	A-CBIL-10-UPenn Mouse PancChip 6.1	*Mus musculus*	Islets and MIN6 murine insulinoma cell line	Islets Pdx-1^+/−^: 3 Islets Pdx-1^+/+^: 3 MIN6 treated: 4 MIN6 control: 4	Sachdeva et al., 2009
GSE12817	[Rat230_2] Affymetrix Rat Genome 230 2.0 Array	*Rattus norvegicus*	Islets	2 mM Glucose: 4 5 mM Glucose: 4 10 mM Glucose: 4 30 mM Glucose: 4	Bensellam et al., 2009
GSE25724	[HG-U133A] Affymetrix Human Genome U133A Array	*Homo sapiens*	Islets	Non-diabetic: 7 Diabetic: 6	Dominguez et al., 2011
GSE20966	[U133_X3P] Affymetrix Human X3P Array	*Homo sapiens*	β-cell-enriched pancreatic tissue	Non-diabetic: 10 Diabetic: 10	Marselli et al., 2010
GSE38642	[HuGene-1_0-st] Affymetrix Human Gene 1.0 ST Array [transcript (gene) version]	*Homo sapiens*	Islets	Non-diabetic: 54 Diabetic: 9	Taneera et al., 2012

### Specificity of ZIP transporter expression within β-cells

Multiple microarray and RNA-seq studies have sought to assess β-cell gene expression relative to other pancreatic cells and additional tissues. Since ZIP paralogues exhibit cell-specific profiles reflecting function [12, 15], β-cell enrichment may indicate important cell-specific roles. Analysis of human islet cell transcriptomics datasets uncovered *SLC39A13* and *SLC39A14* as enriched within β-cells compared to α-cells [2- to 3-fold], and *SLC39A1, SLC39A10* and *SLC39A11* as ≥1.5-fold depleted [36, 37]. However, when β-cell expression was compared to sorted pancreatic exocrine cell populations (human duct and acinar cells), enrichment of *SLC39A7* and *SLC39A9* was observed (1.7- and 1.6-fold respectively) alongside relative depletion of *SLC39A5* (11-fold), *SLC39A8* (4.3-fold), *SLC39A10* (1.8-fold) and *SLC39A11* (1.5-fold) (data calculated from supplementary tables). Similarly RNA-seq data from Nica et al. uncovered depletion of *SLC39A5* and *SLC39A10* within sorted human β-cells over both total islets (2- and 6.8-fold, respectively) and non-β-cells [islet cell populations considered depleted of β-cells (2.8- and 4-fold, respectively)], accompanied by depletion of *SLC39A2* (2-fold over total islets and 4-fold over non-β-cells) and *SLC39A3* (1.7-fold over both total islets and non-β-cells), with enrichment of *SLC39A1* (2.4-fold over total islets and 2.1-fold over non-β-cells), and of *SLC39A14* (1.9-fold, only over non-β-cell preparations) [38].

**Fig. 2:**
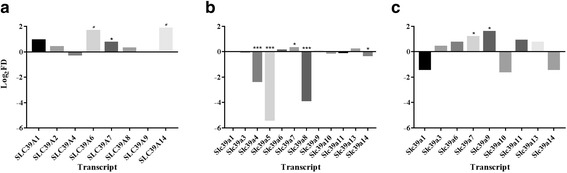
Enrichment of *SLC39A* paralogues within human and murine β-cells. **a** Expression within human β-cell-enriched pancreatic samples compared to 11 other tissues (pancreatic duct cells, cerebrum, kidney, lung, myocardial, skeletal muscle, small intestine, spleen, stomach, testis and thymus). **b** Expression within sorted mouse β-cells compared to mouse islets. **c** Expression within sorted mouse β-cells compared to six other cell types (brain, liver, lung fibroblasts, NPC, skeletal muscle, islets), after exclusion of β-cell depleted paralogues. Data for (**a**) analysed from dataset GSE30803 and (**b**-**c**) analysed from supplementary tables within [44]. ^#^ *P* < 0.15, *P < 0.05, ***P < 0.001. NPC = neural progenitor cells

Analysis of microarray datasets of human β-cell-enriched pancreatic samples and 15 other tissues [β-cell-enriched pancreas, pancreatic duct cells, cerebrum, colon, foetal brain, kidney, liver, lung, myocardial, skeletal muscle, prostate, small intestine, spleen, stomach, testis and thymus (dataset GSE30803)] revealed ≥1.5-fold enrichment of *SLC39A1*, *SLC39A6, SLC39A7* and *SLC39A14*, however without statistical significance [39]. Further investigation of probe-specific expression revealed that relative enrichment was biased by elevated expression of specific paralogues within other tissues (ZIP6 within the brain [40] and prostate [41], ZIP7 within the colon [42] and ZIP14 within the liver [43]). Omitting these tissues indicated ≥3-fold enrichment of *SLC39A6* (q < 0.1) and *SLC39A14* (q < 0.15)*,* and 1.6-fold *SLC39A7* (q < 0.05) enrichment within β-cells compared to the remaining tissues analysed (Fig. 2a).

Analysis of a mouse RNA-seq dataset [44] suggested *Slc39a4*, *Slc39a5* and *Slc39a8* are ≥4-fold depleted within sorted β-cells over islets (Fig. 2b). Further investigation of non-β-cell depleted paralogues compared to total islets and six other cell types [brain, liver, lung fibroblasts, neural progenitor cells (NPC), skeletal muscle, total islet (Fig. 2c)] revealed *Slc39a7* and *Slc39a9* as the most β-cell enriched Zip paralogues in mouse with 2.3- and 3-fold elevated expression, respectively. Analysis of a further rat dataset comparing expression of β-cells over α-cells (dataset GSE13381) displayed ≥1.5-fold enrichment of *Slc39a3* and *Slc39a6*, and ≥2-fold enrichment of *Slc39a7* and *Slc39a14*, but without statistical significance. However, there was no differential *Slc39a* expression between murine islets and five other tissues (adipose, gastrocnemius muscle, hypothalamus, liver, and soleus muscle) from 10-week old lean and obese C57BL/6 and BTBR mice (dataset GSE10785).

### Cytokine stimulation and ZIP transporter expression

Pro-inflammatory cytokines profoundly affect cellular metabolism and utilisation of nutrients such as metal ions [45]. Chronic exposure of islets to the inflammatory cytokines interleukin-1 beta (IL-1β), tumor necrosis factor-alpha (TNF-α) and interferon-gamma (IFN-γ) is associated with β-cell destruction and decreased secretory parameters in both Type 1 and Type 2 Diabetes [46]. Cytokine-dependent expression may indicate ZIP paralogues important for maintaining normal β-cell parameters when adapting to extracellular cytokine stress. RNA-seq dataset analysis of human islets exposed to IL-1β and IFN-γ for two days (dataset GSE35296) revealed 1.4- and 2.0-fold upregulation of *SLC39A8* and *SLC39A14* transcripts, respectively, and 2-fold downregulation of *SLC39A10* [47]. However an additional microarray study using human islets [48] did not show any ZIP transporter transcripts differentially expressed following 48 h incubation with IL-1β and IFN-γ.

Within independent studies, both fluorescence assisted cell sorting (FACS) purified rat β-cells [49] and the rat INS-1E β-cell line [50] were cultured with IL-1β and IFN-γ before microarray analysis (data from both studies calculated from supplementary data). Within rat β-cells [49] differential regulation of *Slc39a1* (2.5-fold) was observed at 2 h, and of *Slc39a1* and *Slc39a10* at both 12 h (2.0- and −3.5-fold, respectively) and 24 h (1.5-fold and −1.6-fold, respectively). Similarly, INS-1E cells [50] displayed upregulation of *Slc39a1* at both 6 and 24 h (2.6- and 2.1-fold, respectively) and of *Slc39a6* at 24 h (1.7-fold), alongside downregulation of *Slc39a13* at 6 h (−1.5-fold). INS-1E cells were additionally analysed after 6 and 24 h incubation with IFN-γ and TNF-α to show *Slc39a1* upregulation (2.8- and 2.4-fold, respectively) and *Slc39a14* downregulation (2.8- and 2.4-fold, respectively).

### PDX-1 and ZIP transporter expression

**Fig. 3:**
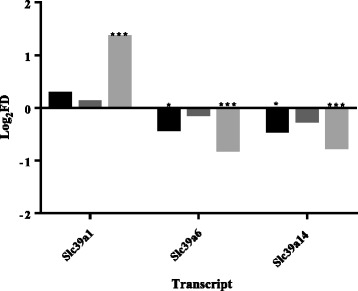
*Slc39a* transcripts differentially expressed in rat INS-1ab cells following PDX-1 overexpression and IL-1β stimulation. Black bars: PDX-1 overexpression; dark grey bars: IL-1β stimulation; light grey bars: PDX-1 overexpression and IL-1β stimulation. Data analysed from dataset GSE40642. *P < 0.05, ***P < 0.001

PDX-1 is the key transcription factor mediating β-cell-specific gene expression within developing and mature β-cells [51]. Changes in ZIP expression as a consequence of PDX-1 activity may indicate roles of respective transporters in maintaining normal β-cell parameters. Critically, constitutive overexpression of PDX-1 sensitises β-cells to cytokine-induced apoptosis [52, 53]. Overexpression of PDX-1 within rat INS-1ab cells (dataset GSE40642) resulted in downregulation of *Slc39a6* and *Slc39a14* (Fig. 3). Stimulation of PDX-1 overexpressing cells with cytokine IL-1β further exacerbated these effects and also upregulated *Slc39a1* ≥ 2.5-fold [54] (Fig. 3). Whereas within rat islets (dataset GSE49786), PDX-1 overexpression upregulated *Slc39a8* (2.1-fold, *P* < 0.01) [55]; however *Slc39a5* and *Slc39a8* were up- and downregulated 1.5- and −2.6-fold, respectively, in mouse MIN6 cells (dataset E-MTAB-127) [56]. Data analysis from E-MTAB-127 additionally showed 2.8-fold downregulation of *Slc39a5* in PDX^+/−^ mouse islets compared to PDX^+/+^ control mouse islets.

### Expression in response to glucose stimulation

Hyperglycaemia is universal within all prediabetic and diabetic cases, and glucose-responsive expression may indicate genes and pathways important for adapting to an enhanced demand for insulin secretion. Examination of microarray datasets of isolated rat islets cultured with 2 mM, 5 mM, 10 mM and 30 mM glucose (dataset GSE12817) uncovered ≥1.5-fold upregulation of *Slc39a2, Slc39a4* and *Slc39a6* and ≥1.5-fold downregulation of *Slc39a3* and *Slc39a5* when glucose increases [57].

### Expression within islets from type 2 diabetic patients

**Fig. 4:**
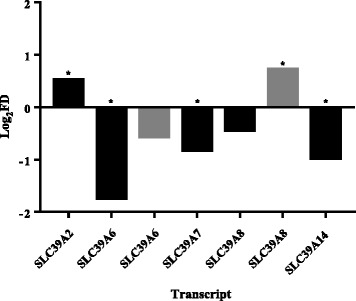
*SLC39A* transcripts differentially expressed in human type 2 diabetic islets compared to non-diabetic islets. Probes targeting the same transcript(s) are averaged for each assay. Black bars: data analysed from dataset GSE25724; grey bars: data analysed from dataset GSE20966. *P < 0.05

Transcriptomic datasets of islets derived from normoglycaemic and Type 2 diabetic patients were next analysed to explore relevance to the human disease. Three paralogues (*SLC39A2*, *SLC39A5* and *SLC39A8*) showed ≥1.5-fold upregulation and four paralogues (*SLC39A6*, *SLC39A7*, *SLC39A8* and *SLC39A14*) ≥1.5-fold downregulation in diabetic compared to non-diabetic individuals [results combined from studies GSE25724 [58] and GSE20966 [59], Fig. 4]. However in a cohort of Nordic patients (dataset GSE38642), no ZIP paralogues were found differentially (≥1.5-fold) expressed between diabetic and non-diabetic islets [60].

### SLC39A Paralogues identified within our systematic review are experimentally verified to show high β-cell/islet expression and sequence conservation

**Fig. 5:**
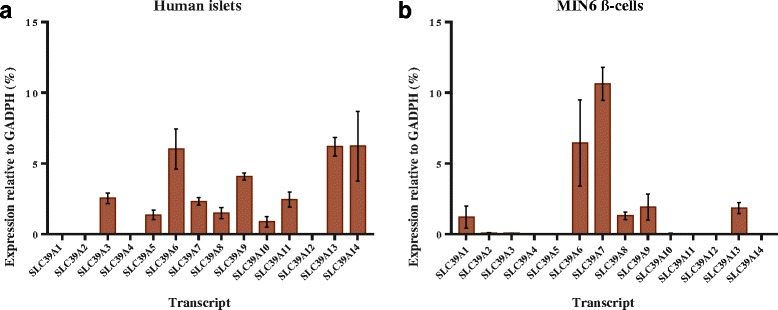
Expression profiles of *SLC39A* mRNA transcripts in human islets and murine MIN6 cells. **a** The human *SLC39A* transcriptome. *N* = 2 and error bars show ± range. **b** The MIN6 β-cell *Slc39a* transcriptome. *N* = 3 and error bars show ±SEM

Enhanced relative expression may suggest a biological relevance of respective putative ZIP orthologues in maintaining intracellular Zn^2+^ homeostasis in β-cells/islets. To verify the biological relevance of the ZIP orthologues we identified in our systematic review in terms of β-cell function, we performed qPCR expression profiling of human and mouse *SLC39A* mRNA transcripts. There was notably a wider range of mRNA for ZIP transporters expressed in human islets, compared with murine MIN6 cells. We observed highest expression of *SLC39A6, SLC39A9, SLC39A13* and *SLC39A14* in human islets and of *Slc39a6* and *Slc39a7* in mouse MIN6 cells (Fig. 5).

We next assessed cross-species homologies between human and putative rodent ZIP orthologues. Enhanced similarity increases the likelihood of inferring molecular function [61], and increases confidence when applying results encompassing data derived from multiple species. We calculated the transcriptomic and proteomic homologies between human and mouse ZIP orthologues, and between mouse and rat ZIP orthologues through bioinformatics. We showed all respective orthologues have high similarities, with lowest protein homology between human and mouse observed for ZIP4 (73%) and highest for ZIP1 (94%). All rat and mouse orthologues showed protein similarities of ≥90% aside from ZIP4 (89.8%) and ZIP14 (89.5%) (Table 2). The high sequence similarities observed indicate that all ZIP paralogues identified as important in our systematic review have the potential to substitute functionally in human, mouse and rat β-cells.

**Table 2:** Percentage similarity between human, mouse and rat ZIP/*SLC39A* protein and mRNA sequences

Gene	Refseq transcript ID			Entrez protein ID			Percentage similarities (human:mouse)		Percentage similarities (mouse:rat)	
	Human	Mouse	Rat	Human	Mouse	Rat	Transcript	Protein	Transcript	Protein
*SLC39A1*	NM_014437.4	NM_013901.2	NM_001134577.1	Q9NY26	Q9QZ03	B5DEF5	80.77	93.83	91.60	98.46
*SLC39A2*	NM_014579.3	NM_001039676.2	NM_001107260.1	Q9NP94	Q2HIZ9	D3ZIN1	78.51	77.99	91.87	94.82
*SLC39A3*	NM_144564.4	NM_134135.1	NM_001008356.1	Q9BRY0	Q99K24	Q5U1X7	80.99	84.04	91.23	97.16
*SLC39A4*	NM_017767.2	NM_028064.2	NM_001077669.1	Q6P5W5	Q78IQ7	A0JPN2	74.55	73.17	91.98	89.79
*SLC39A5*	NM_173596.2	NM_028051.3	NM_001108728.1	Q6ZMH5	Q9D856	D3ZSF7	83.42	84.30	92.24	94.00
*SLC39A6*	NM_012319.3	NM_139143.3	NM_001024745.1	Q13433	Q8C145	Q4V887	80.04	88.20	92.01	95.28
*SLC39A7*	NM_006979.2	NM_008202.2	NM_001164744.1	Q92504	Q31125	Q6MGB4	81.72	85.90	90.62	93.72
*SLC39A8*	NM_022154.5	NM_001135150.1	NM_001011952.1	Q9C0K1	Q91W10	Q5FVQ0	76.15	89.35	90.74	96.10
*SLC39A9*	NM_018375.4	NM_026244.2	NM_001034929.1	Q9NUM3	Q8BFU1	Q3KR82	78.04	93.49	93.35	92.33
*SLC39A10*	NM_001127257.1	NM_172653.2	NM_001108796.2	Q9ULF5	Q6P5F6	D4A517	84.50	87.36	92.25	96.16
*SLC39A11*	NM_001159770.1	NM_001166503.1	NM_001013042.1	Q8N1S5	Q8BWY7	Q6P6S2	77.62	90.32	91.32	95.22
*SLC39A12*	NM_001145195.1	NM_001012305.2	XM_006254285.3	Q504Y0	Q5FWH7	D4A8R5	78.31	78.17	91.15	90.41
*SLC39A13*	NM_001128225.2	NM_001290765.1	NM_001039196.1	Q96H72	Q8BZH0	Q2M1K6	81.54	90.58	93.42	93.84
*SLC39A14*	NM_001128431.2	NM_001135151.1	NM_001107275.1	Q15043	Q75N73	D3ZZM0	75.84	86.91	90.11	89.53

Comparisons were generated using Clustal multiple sequence alignment by MUSCLE (3.8) [34, 35]

## Discussion

Associations between Zn^2+^ status and β-cell function have been extensively described in independent studies [1, 2, 62]. ZnT8 expression is positively correlated with granule Zn^2+^ release and glucose tolerance in mice [63], and high glucose stimulation increases free Zn^2+^ content within mouse islets [22] and hamster HIT-T15 cells [64]. Intracellular Zn^2+^ exhibits roles in protection against oxidative stress-induced apoptosis [65] whereas chronic elevation contributes to β-cell dysfunction [22]. The ZIP importer paralogues responsible for maintaining β-cell Zn^2+^ homeostasis remain largely unexplored and are important to investigate for understanding β-cell function in health and diabetic disease.

A systematic review allows integrated analysis of relative consistencies in differential expression from high throughput gene expression techniques, despite heterogeneities between studies involving experimental design and platform used. It has the capacity to identify consistent but modest variations, important for genes involved in processes where small expression changes can have amplified effects. Through this systematic review we re-analysed raw microarray and RNA-seq data in parallel with unannotated high-throughput datasets to compare and contrast β-cell ZIP complemets in human, mouse and rat β-cells/islets. We show enrichment of mRNA for ZIP7 and ZIP9 within rodent and ZIP6, ZIP7 and ZIP14 within human, with mRNA for ZIP1, ZIP6 and ZIP14 differentially expressed in response to cytokines and PDX-1 within rodent, and ZIP6 in response to diabetic status in human and glucose in rat. To query the biological relevance of our data, we carried out experimental expression profiling of human islet and MIN6 β-cell cDNA, and computationally aligned human, mouse and rat mRNA and protein sequences. Highest expression was observed for mRNA corresponding to ZIP6, ZIP9, ZIP13 and ZIP14 in human islets and ZIP6 and ZIP7 in mouse MIN6 cells, which is in agreement with previous observations [21]. The mRNA profile for ZIPs generated through our qPCR analyses also corresponds well to expression data on specific isoforms in human islets as produced by RNA-seq (Additional file 4). All ZIP orthologues displayed high sequence conservation between species. Surprisingly ZIP4, which is essential for intestinal zinc uptake in both mouse and human [27, 66], shows the lowest homology (73%) between these two species and it also does not appear to play a major role in β-cells. Based on their expression levels, relative enrichment in β-cells/islets (compared with other cells/tissues), and regulation in response to conditions relevant to diabetes, we propose that ZIP6, ZIP7 and ZIP14 in human, and ZIP6 and ZIP7 in rodent may be of particular importance for β-cell Zn^2+^ uptake and/or homeostasis. This conclusion is similar to that of Liu et al. [21], who highlighted the roles of ZIP6 and ZIP7 in β-cell zinc transport and viability. Our study also indentifies ZIP1, ZIP9 and ZIP13 as being of potential additional significance for β-cell function.

The abundance of ZIP transporters varies substantially between tissues and cells, allowing those with differing Zn^2+^ affinities, cellular localisations and regulatory mechanisms to tightly maintain the homeostatic balance [67]. We found significant differences in ZIP mRNA abundance between β-cells and non-pancreatic tissues; specifically, enrichment of ZIP7 and ZIP9 within mouse β-cells, and ZIP6, ZIP7 and ZIP14 within human islets. With the exception for ZIP7, which is found in the endoplasmic reticulum (ER) and in some cells in the Golgi apparatus, these zinc channels are operating at the plasma membrane [13, 68]. ZIP6 and ZIP7 enrichment is consistent with a report [21], suggesting that ZIP6 and ZIP7 mediate influx of zinc into the β-cell cytosol in tandem from the plasma membrane and the ER. In addition to their roles in transporting zinc, ZIP6, ZIP7 and ZIP14 strongly stimulate cell proliferation, drastically increasing the number of cells in G_2_/M phase, and their expression changes in cancers [27, 69–73]. Also of potential importance is that ZIP14 mediates import of both zinc and non-heme iron [74, 75] and that ZIP9 has been identified as a plasma membrane androgen receptor [76]. Interestingly, transcripts of ZIP9 and ZIP14, which were both found expressed at comparable abundances to ZIP6 and ZIP7 within human islets by ourselves and others [21], were additionally enriched within β-cells. ZIP9 and ZIP14 both show predicted localisation at the plasma membrane (with localisation of ZIP9 at the Golgi and trans-Golgi network additionally described) [77, 78], and currently remain unexplored in this context. Our expression profiling further identified ZIP13 as highly expressed in both human islets and MIN6 cells. ZIP13 is phylogenically grouped with ZIP7 [79] and studies have suggested ZIP13 localises at the ER, Golgi [80, 81] and intracellular vesicles [82]. However, to our knowledge ZIP13 has not been studied in β-cells. ZIP9, ZIP13 and ZIP14 may represent novel targets for understanding β-cell zinc uptake and homeostasis.

PDX-1 is the major transcriptional regulator in mature β-cells and mediates expression of key β-cell genes, with homozygous mutations linked to Type 2 Diabetes development [83]. Furthermore, PDX-1 drives β-cell (re)generation from neurogenin-3 positive endocrine precursors and pancreatic α-cells [84, 85], and β-cell-specific recovery of activity within Ins2^Akita^ mice (βPdx1; Ins2^Akita^ mice) promotes significantly improved glucose tolerance compared to control littermates [86]. Of interest, PDX-1 binds enhancers (cis elements) of the ZnT8 gene *SLC30A8* [87], indicating a role of PDX-1 in β-cell zinc homeostasis parallel to its role in insulin gene regulation [88]. Our analysis suggests PDX-1 activity sensitizes the β-cell zinc response to cytokines through ZIP6 and ZIP14 downregulation and ZIP1 upregulation within rat INS-1ab cells. We additionally established ZIP1 to be consistently upregulated following stimulation with IL-1β and IFN-γ, and IFN-γ and TNF-α within rat β-cells [49] and INS-1E cells [50], highlighting ZIP1 as potentially important in the adaptive response to cytokines. Interestingly, ZIP1 and ZIP6 abundances have been negatively correlated with the obesity-associated inflammatory state [89]. In contrast to the data in rodents, our review further identified ZIP8 and ZIP14 upregulation in response to the inflammatory cytokines IL-1β and IFN-γ in human islets [47]. Inflammatory mediators such as lipopolysaccharides (LPS) and TNF-α upregulate *SLC39A8* within human lung epithelia [90] and *Slc39a14* is upregulated in response to LPS-initiated inflammation within the mouse pancreas and liver [43] and shows an acute-phase gene response to IL-6 [91].

Hyperglycaemia is well recognised as a universal driver in the pathogenesis of Type 2 Diabetes [92]. Our analysis showed high glucose stimulation of rat islets significantly enhanced ZIP6 mRNA expression, consistent with glucose-dependent increases of additional ZIP7 and ZIP8 upregulation [22]. Similarly, analysis of islets from human type 2 diabetic donors displayed ZIP6, ZIP7, ZIP8 and ZIP14 mRNA downregulation compared to normoglycaemic controls [58, 59]. Decreased transcript expression supports a disease relevance of these paralogues for mediating β-cell zinc accumulation, indicating abnormally low zinc uptake may occur within diabetic β-cells as a result of disrupted ZIP6, ZIP7, ZIP8 and/or ZIP14 expression.

At a proteomic level no significant differences in protein abundances were observed for any ZIP paralogue within human islets incubated with high or low glucose [93–95]. Though in one of these studies non-significant trends for enrichment of ZIP6 (2.6-fold) and ZIP14 (1.6-fold) in human islets were observed following culture in high compared to low glucose [95]. However, these proteomic studies likely bias towards proteins with higher abundances [96], and accurately evaluating less abundant species away from central pathways and those in complexes remains challenging, with membrane proteins imposing further challenges [97]. Although it is acknowledged that mRNA abundances often poorly correlate with protein abundances and functional activity [98], transcriptomic analysis remains important for pinpointing expression control and pathways of disruption during disease states.

This systematic review provides an overview of ZIP transcript expression in the context of β-cell specificity, cytokine stimulation, PDX-1 activity, glucose status and Type 2 Diabetes. It has allowed us to collectively analyse ZIP expression within multiple high throughput datasets, complemented by experimental work, providing evidence for differential regulation as a consequence of β-cell stresses associated with decreased insulin secretion. The study’s limitations should nevertheless be acknowledged. Firstly, although all raw datasets (if appropriate) were subjected to the same normalisation process to minimise inconsistencies, the platform and genomic heterogeneities and differing probe hybridization efficiencies could skew global interpretation, and the analysis used may not have been equally suited to all datasets. Secondly, RNA-seq offers unbiased analysis of sequences present however microarray datasets are limited by hybridization efficiencies and the probes present [99], such that in multiple studies probes did not target all ZIP paralogues. Multiple datasets analysed and our qPCR expression data utilised islets incorporating non-β-cells. Relative *SLC39A* abundances may be impacted by additional cell populations, such as *SLC39A14* enrichment in α-cells [100]. Furthermore, although we have shown high conservation of ZIP mRNA and protein sequences between human and mouse, results may not be entirely translatable across species. Finally, several microarray studies identified within the systematic review search criteria were excluded during the final screening due to the absence of available experimental data for download and analysis. Despite these limitations, our systematic review distinguishes specific *SLC39A* paralogues as important within each human and rodent β-cells. The results are strongly supported by our experimental expression profiling of human islet and MIN6 β-cell cDNA through confirming relative enrichment and a biological relevance.

## Conclusions

**Fig. 6:**
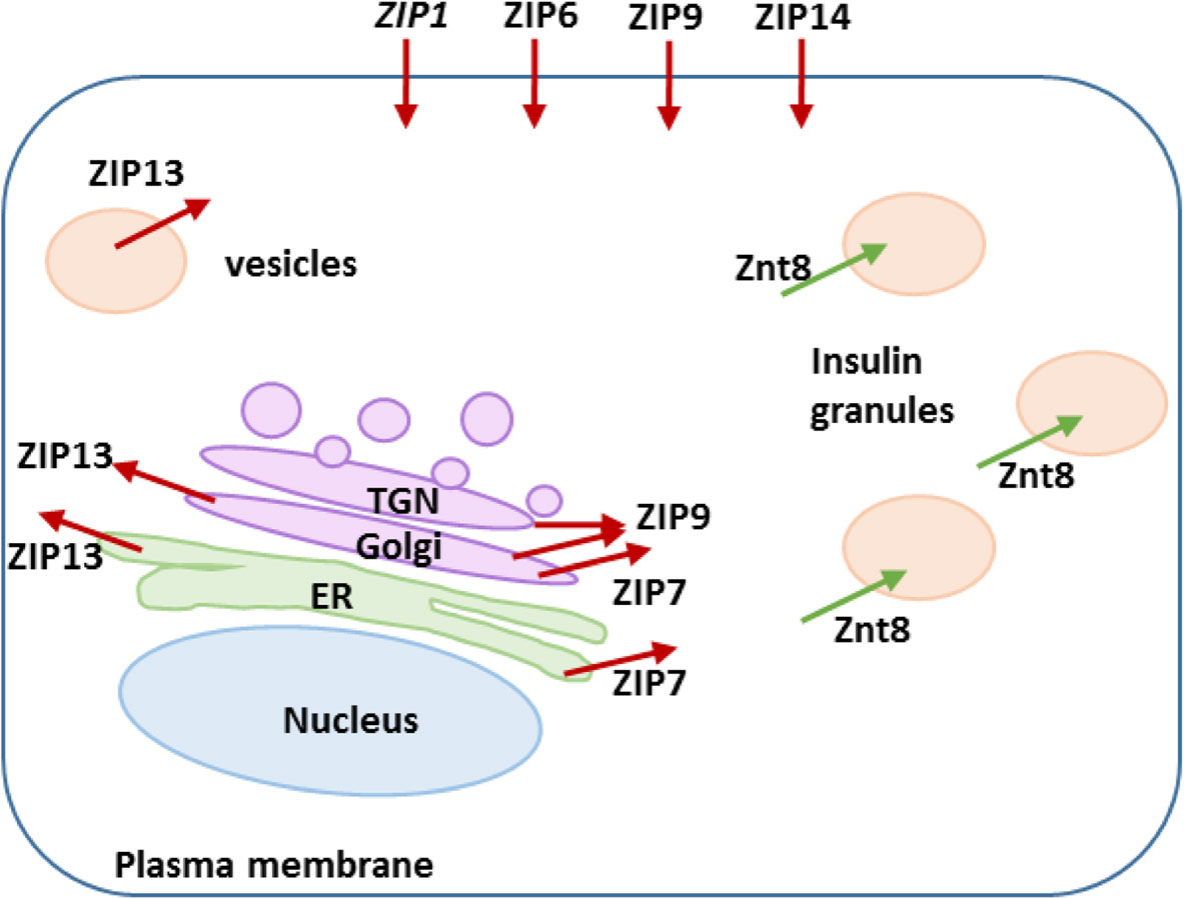
Predicted subcellular localisation of the identified ZIP transporters within human and rodent β-cells. Based on the data analysed in the present study ZIP6, ZIP7, ZIP9, ZIP13 and ZIP14 in human, and the same transporters in addition to ZIP1 in rodent appear to be of particular importance for β-cell biology and pathology. Zinc Transporter 8 (ZnT8) which transports zinc into the insulin granules is also shown. ER = endoplasmic reticulum; TGN = trans-Golgi network

We have used a systematic approach to identify key ZIP complements in human, mouse and rat β-cells. We have verified a biological importance of these paralogues through proving high relative expression in human islets and/or murine MIN6 β-cells, and have demonstrated their potential to serve as functional orthologues in human and rodent through verifying high sequence similarities. Importantly, our results highlight similarities and potentially biologically relevant differences in zinc regulation between human and rodent ZIP orthologues which may prove critical when evaluating rodent β-cell models of disease. We propose ZIP6 and ZIP7 serve as key functional rodent-human orthologues in β-cells. We further identify ZIP9, ZIP13 and ZIP14 in human and rodent, and ZIP1 in rodent as potentially biologically important for β-cell function (Fig. 6). These paralogues represent interesting targets for future investigation into zinc regulation and homeostasis in β-cell failure and Type 2 Diabetes.

## Additional files

Additional file 1: Analysed datasets. (XLSX 61 kb)

Additional file 2: Table S1. Designs for human qPCR assays undertaken. (DOCX 12 kb)

Additional file 3: Table S2. Designs for mouse qPCR assays undertaken. (DOCX 12 kb)

Additional file 4: ZIP isoforms in human islets. (DOCX 56 kb)

## Abbreviations

ER: Endoplasmic reticulum
FACS: Fluorescence assisted cell sorting
FD: Fold difference
FDR: False Discovery Rate
GEO: Gene Expression Omnibus
IFN-γ: Interferon-gamma
IL-1β: Interleukin-1 beta
Log_2_FD: Log_2_ transformed FD
LPS: Lipopolysaccharides
NPC: Neural progenitor cells
PDX-1: Pancreatic and duodenal homeobox 1
qPCR: quantitative PCR
TNF-α: Tumor necrosis factor-alpha
UPL: Universal Probe Library
ZIP: Zrt- and Irt-like protein
Zn^2+^: Zinc ions
ZnT8: Zinc transporter 8
