# Supplementary material for: Expression of the ZIP/SLC39A transporters in β-cells: a systematic review and integration of multiple datasets
Source: BMC Genomics. 2017 Sep 11;18:719. doi: 10.1186/s12864-017-4119-2 (PMC5594519; doi:10.1186/s12864-017-4119-2)
Supplement: Supplementary file 2 — Designs for human qPCR assays undertaken. (DOCX 12 kb) [file 12864_2017_4119_MOESM2_ESM.docx]

**Additional file 2: Table S1.** Designs for human qPCR assays undertaken.

| **Gene** | **RefSeq ID** | **Forward primer (5’ – 3’)** | **Reverse primer (5’ – 3’)** | **Amplicon** | **UPL Probe** |
| --- | --- | --- | --- | --- | --- |
| *SLC39A1* | NM_014437.3 | GGAGAAAGCTCCGGGAAA | GAGTGACGTCAGTTAGGAGCAA | 91 | #71 |
| *SLC39A2* | NM_014579.3 | GAACAGATCAGCAAGTGAGAGAAA | AGCTCTCCATAGGGATACTCCA | 75 | #09 |
| *SLC39A3* | NM_144564.4 | GTTTCTGGCCACGTGCTT | AGGCTCAGGACCTTCTGGA | 69 | #74 |
| *SLC39A4* | NM_017767.2 | GCTCCAGTGTGTGGGACA | GCCTGTTCCGACAGTCCA | 73 | #46 |
| *SLC39A5* | NM_173596.2 | CCTCTTCCTGCTCTTTGTGC | TCGAGATTCCTTCGTTTTCG | 96 | #45 |
| *SLC39A6* | NM_012319.3 | ACTGGCCGTTGGGACTTT | ATGGTGGTGACTTGCATGAG | 73 | #09 |
| *SLC39A7* | NM_006979.2 | CGAAGGTGGAACGGAACTT | AGGCCTGGAAAGGATGGTAG | 134 | #55 |
| *SLC39A8* | NM_022154.5 | TTTTGGTGGGCAACAATTTC | CAGCATATCATTCATCTCTGGAA | 107 | #67 |
| *SLC39A9* | NM_018375.4 | GGTCTGGTTGTCCATGCTG | AACTGGACACTGGTCTGTGAAGT | 77 | #32 |
| *SLC39A10* | NM_001127257.1 | TGTAGCCTTGGTGGATATGCT | CCACAGGACAAAAGCCATGT | 77 | #09 |
| *SLC39A11* | NM_139177.3 | GGGCTGATGGAAGTGCAG | CCTTGGAGCATGCTGGATAC | 63 | #19 |
| *SLC39A12* | NM_152725.3 | TGAAATACTCTCCAGGATTTAAAAGG | GGGGTAAACTTGTAACCAAAGGA | 96 | #50 |
| *SLC39A13* | NM_001128225.2 | GGCCAACACCATCGATAACT | TTGTCAGGAGCCCGATCTT | 83 | #05 |
| *SLC39A14* | NM_015359.4 | TCTCTGCCAACTGGATTTTTG | CCTCATTCATCTCAGGGAACA | 84 | #78 |
| *UBC* | M26880.1/M26880.EMI | CAGAGGTTGATCTTTGCTGGA | GCAGGGTGGACTCTTTCTGA | 82 | #11 |
| *GAPDH* | NM_002046.3 | AGCCACATCGCTCAGACAC | GCCCAATACGACCAAATCC | 66 | #60 |
