# Supplementary material for: Expression of the ZIP/SLC39A transporters in β-cells: a systematic review and integration of multiple datasets
Source: BMC Genomics. 2017 Sep 11;18:719. doi: 10.1186/s12864-017-4119-2 (PMC5594519; doi:10.1186/s12864-017-4119-2)
Supplement: Supplementary file 3 — Designs for mouse qPCR assays undertaken. (DOCX 12 kb) [file 12864_2017_4119_MOESM3_ESM.docx]

**Additional file 3: Table S2.** Designs for mouse qPCR assays undertaken.

| **Gene** | **RefSeq ID** | **Forward primer (5’ – 3’)** | **Reverse primer (5’ – 3’)** | **Amplicon** | **UPL Probe** |
| --- | --- | --- | --- | --- | --- |
| *Slc39a1* | NM_013901.2 | CGACAGCAATGGAGTGAGAC | TCCGATGCGACTGCTTCT | 119 | #81 |
| *Slc39a2* | NM_001039676.2 | CCTGCTTGCTCTTCTGGTTC | ACCCTGTGGTGATGACCTG | 99 | #26 |
| *Slc39a3* | NM_134135.1 | CGTATTCCTGGCTACATGCTT | CGGGTAGTCGGTGCTGAT | 97 | #27 |
| *Slc39a4* | NM_028064.2 | CAGCTACTGCAGAAGATTGAGG | TCCAGCAGTTGGGGAAGAT | 83 | #07 |
| *Slc39a5* | NM_028051.3 | GGCTGACCATCTGAATGAGG | AGGGCTCAGTCCAAAATTGA | 67 | #33 |
| *Slc39a6* | NM_139143.3 | CCAGTCCCTTCGGACCTC | CTGTGGCCATTGCACCTT | 89 | #70 |
| *Slc39a7* | NM_008202.2 | GGATTTTGCCATCCTGGTC | TTGCAGTCACGAGTTGCAG | 71 | #71 |
| *Slc39a8* | NM_026228.4 | TCAGCGTTGTATCCCTCCA | GTTTGGGCCCCTTCAGAC | 69 | #21 |
| *Slc39a9* | NM_026244.2 | CGTGGCAATAATGCTACACAA | CATGCATCAGGAAGGAAACC | 62 | #67 |
| *Slc39a10* | NM_172653.2 | TTTCAGATCATAAGTTAAACAGCACA | CCGAGTCATCCGTTCCAG | 78 | #89 |
| *Slc39a11* | NM_027216.5 | GAGGATTGCTTTGCTCATCC | AATCCTACGCCAACAGCAAG | 72 | #09 |
| *Slc39a12* | NM_001012305.2 | TGGACACAAGGAGACTGCAA | TTCCCCCAGCTGTGAGTAAC | 64 | #38 |
| *Slc39a13* | NM_026721.2 | AGATGTTCCTCAACAGCAAGG | GCAGCAGTGGGGTCTTTG | 61 | #20 |
| *Slc39a14* | NM_001135151.1 | GCTCTCTAACGCCCTTTTCC | ATTGTCCTGAGGGTTGAAGC | 61 | #110 |
| *Ubc* | NM_019639.4 | GACCAGCAGCAGGCTGATCTT | CCTCTGAGGCGAAGGACTAA | 110 | #11 |
| *Gapdh* | NM_008084.2 | TGTCCGTCGTGGATCTGC | CCTGCTTCACCACCTTCTTG | 75 | #80 |
