# Supplementary material for: Expression of the ZIP/SLC39A transporters in β-cells: a systematic review and integration of multiple datasets
Source: BMC Genomics. 2017 Sep 11;18:719. doi: 10.1186/s12864-017-4119-2 (PMC5594519; doi:10.1186/s12864-017-4119-2)
Supplement: Supplementary file 4 — ZIP isoforms in human islets. (DOCX 56 kb) [file 12864_2017_4119_MOESM4_ESM.docx]

**Additional file 4. ZIP isoforms in human islets.** Data produced through RNAseq from Eizirik DL et al., PLoS Genet. 2012;8(3):e1002552.
